# CHRODIS criteria applied to the MASK (MACVIA-ARIA Sentinel NetworK) Good Practice in allergic rhinitis: a SUNFRAIL report

**DOI:** 10.1186/s13601-017-0173-8

**Published:** 2017-10-23

**Authors:** J. Bousquet, G. L. Onorato, C. Bachert, M. Barbolini, A. Bedbrook, L. Bjermer, J. Correia de Sousa, N. H. Chavannes, A. A. Cruz, E. De Manuel Keenoy, P. Devillier, J. Fonseca, S. Hun, T. Kostka, P. W. Hellings, M. Illario, J. C. Ivancevich, D. Larenas-Linnemann, J. Millot-Keurinck, D. Ryan, B. Samolinski, A. Sheikh, A. Yorgancioglu, I. Agache, S. Arnavielhe, M. Bewick, I. Annesi-Maesano, J. M. Anto, K. C. Bergmann, C. Bindslev-Jensen, S. Bosnic-Anticevich, J. Bouchard, D. P. Caimmi, P. Camargos, G. W. Canonica, V. Cardona, A. M. Carriazo, C. Cingi, E. Colgan, A. Custovic, R. Dahl, P. Demoly, G. De Vries, W. J. Fokkens, J. F. Fontaine, B. Gemicioğlu, N. Guldemond, Z. Gutter, T. Haahtela, B. Hellqvist-Dahl, E. Jares, G. Joos, J. Just, N. Khaltaev, T. Keil, L. Klimek, M. L. Kowalski, I. Kull, P. Kuna, V. Kvedariene, D. Laune, R. Louis, A. Magnan, J. Malva, E. Mathieu-Dupas, E. Melén, E. Menditto, M. Morais-Almeida, R. Mösges, J. Mullol, R. Murray, H. Neffen, R. O’Hehir, S. Palkonen, N. G. Papadopoulos, G. Passalacqua, J. L. Pépin, F. Portejoie, D. Price, B. Pugin, F. Raciborski, F. E. R. Simons, M. Sova, O. Spranger, C. Stellato, A. Todo Bom, P. V. Tomazic, M. Triggiani, A. Valero, E. Valovirta, O. VandenPlas, A. Valiulis, M. van Eerd, M. T. Ventura, M. Wickman, I. Young, T. Zuberbier, A. Zurkuhlen, A. Senn

**Affiliations:** 1 Contre les MAladies Chroniques pour un VIeillissement Actif en France European Innovation Partnership on Active and Healthy Ageing Reference Site, Montpellier, France; 2 INSERM U 1168, VIMA: Ageing and Chronic Diseases Epidemiological and Public Health Approaches, Villejuif, Université Versailles St-Quentin-en-Yvelines, UMR-S 1168, Montigny le Bretonneux, France; 3 Upper Airways Research Laboratory, ENT Department, Ghent University Hospital, Ghent, Belgium; 4 Regione Emilia Romagna - Agenzia Sanitaria e Sociale, Reference Site of the European Innovation Partnership on Healthy and Active Ageing, Bologna, Italy; 5 Department of Respiratory Medicine and Allergology, University Hospital, Lund, Sweden; 6 Life and Health Sciences Research Institute (ICVS), School of Health Sciences, University of Minho, Braga, Portugal; 7 Department of Public Health and Primary Care, Leiden University Medical Center, Leiden, The Netherlands; 8 ProAR – Nucleo de Excelencia em Asma, Federal University of Bahia, Salvador, Brazil; 9 GARD Executive Committee, Salvador, Brazil; 10 Kronikgune, International Centre of Excellence in Chronicity Research Barakaldo, Bizkaia Bilbao, Spain; 11 Laboratoire de Pharmacologie Respiratoire UPRES EA220, Pôle des Maladies Respiratoires, Hôpital Foch, Suresnes Université Versailles Saint-Quentin, Versailles, France; 12 Center for Health Technology and Services Research- CINTESIS, Faculdade de Medicina, Universidade do Porto, Porto, Portugal; 13 Allergy Unit, CUF Porto Instituto and Hospital, Porto, Portugal; 14 Public Health Agency Northern Ireland, Belfast, UK; 15 Department of Geriatrics, Medical University of Lodz, Healthy Ageing Research Centre (HARC), Lodz, Poland; 16 Laboratory of Clinical Immunology, Department of Microbiology and Immunology, KU Leuven, Louvain, Belgium; 17 Division for Health Innovation, Campania Region and Federico II University Hospital Naples (R&D and DISMET), Naples, Italy; 18 Allergy and Immunology Department, Santa Isabel, Buenos Aires, Argentina; 19 Clínica de Alergia, Asma y Pediatría, Hospital Médica Sur, Mexico, Mexico; 20 Caisse Assurance Retraite et Santé Au Travail Languedoc-Roussillon (CARSAT-LR), 34000 Montpellier, France; 21 Allergy and Respiratory Research Group, Usher Institute of Population Health Sciences and Informatics, University of Edinburgh, Edinburgh, UK; 22 Department of Prevention of Envinronmental Hazards and Allergology, Medical University of Warsaw, Warsaw, Poland; 23 Asthma UK Centre for Applied Research, Centre of Medical Informatics, Usher Institute of Population Health Sciences and Informatics, The University of Edinburgh, Edinburgh, UK; 24 Department of Pulmonology, Celal Bayar University, Manisa, Turkey; 25 GARD Executive Committee, Manisa, Turkey; 26 Faculty of Medicine, Transylvania University, Brasov, Romania; 27 Kyomed, Montpellier, France; 28 iQ4U Consultants Ltd, London, UK; 29 EPAR U707 INSERM, Paris and EPAR UMR-S UPMC, Paris VI, Paris, France; 30 Centre for Research in Environmental Epidemiology (CREAL), ISGLoBAL, Barcelona, Spain; 31 IMIM (Hospital del Mar Research Institute), Barcelona, Spain; 32 CIBER Epidemiología y Salud Pública (CIBERESP), Madrid, Spain; 33 Universitat Pompeu Fabra (UPF), Barcelona, Spain; 34 Comprehensive Allergy-Centre-Charité, Department of Dermatology and Allergy, Charité - Universitätsmedizin Berlin, Berlin, Germany; 35 Global Allergy and Asthma European Network (GA2LEN), Berlin, Germany; 36 Department of Dermatology and Allergy Centre, Odense University Hospital, Odense, Denmark; 37 Woolcock Institute of Medical Research, University of Sydney and Sydney Local Health District, Glebe, NSW Australia; 38 Laval’s University, Quebec City, Canada; 39 Hôpital de la Malbaie, Quebec City, Canada; 40 CHRU de Montpellier, Sorbonne Universités, UPMC Paris 06, UMR-S 1136, IPLESP, Equipe EPAR, 75013 Paris, France; 41 Department of Respiratory Diseases, Montpellier University Hospital, Montpellier, France; 42 Department of Pediatrics, Medical School, Federal University of Minas Gerais, Belo Horizonte, Brazil; 43 Personalized Medicine Clinic Asthma and Allergy, Humanitas University, Humanitas Research Hospital, Rozzano, Milan Italy; 44 Allergologia, S Medicina Interna, Hospital Vall d’Hebron, Barcelona, Spain; 45 Regional Ministry of Health of Andalusia, Seville, Spain; 46 ENT Department, Medical Faculty, Eskisehir Osmangazi University, Eskisehir, Turkey; 47 Department of Health, Social Services and Public Safety, Belfast, Northern Ireland, UK; 48 Department of Pediatric, Imperial College London, London, UK; 49 Peercode DV, Gerdermalsen, The Netherlands; 50 Department of Otorhinolaryngology, Academic Medical Centre, Amsterdam, The Netherlands; 51 Allergist, Reims, France; 52 Department of Pulmonary Diseases, Cerrahpasa Faculty of Medicine, Istanbul University, Istanbul, Turkey; 53 Institute of Health Policy and Management iBMG, Erasmus University, Rotterdam, The Netherlands; 54 University Hospital Olomouc – National eHealth Centre, Olomouc, Czech Republic; 55 Skin and Allergy Hospital, Helsinki University Hospital, Helsinki, Finland; 56 Department of Respiratory Diseases, Odense University Hospital, Odense, Denmark; 57 Libra Foundation, Buenos Aires, Argentina; 58 Department of Respiratory Medicine, Ghent University Hospital, Ghent, Belgium; 59 Allergology Department, Centre de l’Asthme et des Allergies Hôpital d’Enfants Armand-Trousseau (APHP), Paris, France; 60 UPMC Univ Paris 06, UMR_S 1136, Institut Pierre Louis d’Epidémiologie et de Santé Publique, Equipe EPAR, Sorbonne Universités, 75013 Paris, France; 61 GARD Chairman, Geneva, Switzerland; 62 Institute of Social Medicine, Epidemiology and Health Economics, Charité - Universitätsmedizin Berlin, Berlin, Germany; 63 Institute for Clinical Epidemiology and Biometry, University of Wuerzburg, Würzburg, Germany; 64 Center for Rhinology and Allergology, Wiesbaden, Germany; 65 Department of Immunology, Rheumatology and Allergy, Medical University of Lodz, and HARC, Lodz, Poland; 66 Department of Clinical Science and Education, Södersjukhuset, Karolinska Institutet Stockholm, Stockholm, Sweden; 67 Division of Internal Medicine, Asthma and Allergy, Barlicki University Hospital, Medical University of Lodz, Lodz, Poland; 68 Clinic of Infectious, Chest Diseases, Dermatology and Allergology, Vilnius University, Vilnius, Lithuania; 69 Department of Pulmonary Medicine, CHU Sart-Tilman, Liege, Belgium; 70 Service de Pneumologie, UMR INSERM, UMR1087 and CNR 6291, l’institut du thorax, University of Nantes, Nantes, France; 71 Institute of Biomedical Imaging and Life Sciences (IBILI), Faculty of Medicine, University of Coimbra, Coimbra, Portugal; 72 Ageing@Coimbra EIP-AHA Reference Site, Coimbra, Portugal; 73 Sachs’ Children and Youth Hospital, Södersjukhuset, Stockholm and Institute of Environmental Medicine, Karolinska Institutet, Stockholm, Sweden; 74 CIRFF, Federico II University, Naples, Italy; 75 Allergy and Clinical Immunology Department, Hospital CUF-Descobertas, Lisbon, Portugal; 76 Institute of Medical Statistics, Informatics and Epidemiology, Medical Faculty, University of Cologne, Cologne, Germany; 77 Clinical and Experimental Respiratory Immunoallergy, ENT Department, Hospital Clínic, IDIBAPS, CIBERES, Universitat de Barcelona, Barcelona, Spain; 78 Medical Communications Consultant, MedScript Ltd, Dundalk, Co Louth Ireland; 79 Argentina Center for Allergy and Immunology, Alassia Children’s Hospital, Santa Fe, Santa Fe, Argentina; 80 Department of Allergy, Immunology and Respiratory Medicine, Alfred Hospital and Central Clinical School, Monash University, Melbourne, VIC Australia; 81 Department of Immunology, Monash University, Melbourne, VIC Australia; 82 EFA European Federation of Allergy and Airways Diseases Patients’ Associations, Brussels, Belgium; 83 Center for Pediatrics and Child Health, Institute of Human Development, Royal Manchester Children’s Hospital, University of Manchester, Manchester, M13 9WL UK; 84 Allergy Department, 2nd Pediatric Clinic, Athens General Children’s Hospital “P&A Kyriakou,”, University of Athens, Athens, 11527 Greece; 85 Allergy and Respiratory Diseases, IRCCS San Martino Hospital, IST-University of Genoa, Genoa, Italy; 86 CHU Grenoble, La Tronche, France; 87 Observational and Pragmatic Research Institute, Singapore, Singapore; 88 Optimum Patient Care, Cambridge, UK; 89 Academic Centre of Primary Care, University of Aberdeen, Aberdeen, UK; 90 Department of Pediatrics and Child Health, Department of Immunology, Faculty of Medicine, University of Manitoba, Winnipeg, Manitoba Canada; 91 University Hospital Olomouc, Olomouc, Czech Republic; 92 Global Allergy and Asthma Platform GAAPP, Altgasse 8-10, 1130 Vienna, Austria; 93 Department of Medicine, Surgery and Dentistry “Scuola Medica Salernitana”, University of Salerno, Salerno, Italy; 94 Imunoalergologia, Centro Hospitalar Universitário de Coimbra and Faculty of Medicine, University of Coimbra, Coimbra, Portugal; 95 Department of ENT, Medical University of Graz, Graz, Austria; 96 Pneumology and Allergy Department Hospital Clínic, Clinical and Experimental Respiratory Immunoallergy, IDIBAPS, CIBERES, University of Barcelona, Barcelona, Spain; 97 Department of Chest Medicine, Centre Hospitalier Universitaire UCL Namur, Université Catholique de Louvain, Yvoir, Belgium; 98 Unit of Geriatric Immunoallergology, University of Bari Medical School, Bari, Italy; 99 Queen’s University Belfast, Belfast, Northern Ireland, UK; 100 Gesundheitsregion KölnBonn - HRCB Projekt GmbH, Kohln, Germany; 101 EC-CNECT-H2, European Commission, Brussels, Belgium; 102 CHU Montpellier, 371 Avenue du Doyen Gaston Giraud, 34295 Montpellier Cedex 5, France

**Keywords:** Rhinitis, Asthma, CHRODIS, ARIA, MASK, Sunfrail, Good Practices

## Abstract

A Good Practice is a practice that works well, produces good results, and is recommended as a model. MACVIA-ARIA Sentinel Network (MASK), the new Allergic Rhinitis and its Impact on Asthma (ARIA) initiative, is an example of a Good Practice focusing on the implementation of multi-sectoral care pathways using emerging technologies with real life data in rhinitis and asthma multi-morbidity. The European Union Joint Action on Chronic Diseases and Promoting Healthy Ageing across the Life Cycle (JA-CHRODIS) has developed a checklist of 28 items for the evaluation of Good Practices. SUNFRAIL (Reference Sites Network for Prevention and Care of Frailty and Chronic Conditions in community dwelling persons of EU Countries), a European Union project, assessed whether MASK is in line with the 28 items of JA-CHRODIS. A short summary was proposed for each item and 18 experts, all members of ARIA and SUNFRAIL from 12 countries, assessed the 28 items using a Survey Monkey-based questionnaire. A visual analogue scale (VAS) from 0 (strongly disagree) to 100 (strongly agree) was used. Agreement equal or over 75% was observed for 14 items (50%). MASK is following the JA-CHRODIS recommendations for the evaluation of Good Practices.

## Background

European Innovation Partnerships (EIPs) aim to enhance European Union (EU) competitiveness and tackle societal challenges through research and innovation. To tackle the potential of ageing in the EU, the European Commission-within its Innovation Union policy-launched the European Innovation Partnership on Active and Healthy Ageing, Directorate General for Health and Food Safety, Directorate General for Communications Networks, Content & Technology (EIP on AHA, DG Santé and DG CONNECT) [1]. The B3 Action Plan promotes integrated care models for chronic diseases, including the use of remote monitoring.

The initiative AIRWAYS ICPs (EIP on AHA) is the model of chronic diseases of the B3 Action Plan [2, 3]. It is a GARD (Global Alliance against Chronic Respiratory Diseases, WHO) Research Demonstration Project [4]. AIRWAYS ICPs was initiated in 2013 by the EIP on AHA Reference Site MACVIA-LR (*Contre les MAladies Chroniques pour un VIeillissement Actif en* Languedoc-Roussillon, France) [5]. The aim of AIRWAYS ICPs was to launch a collaboration to develop practical multi-sectoral care pathways (ICPs) in order to: (1) reduce chronic respiratory disease burden, mortality and multi-morbidity; (2) improve education of all stakeholders; (3) improve work productivity; (4) promote AHA; and (5) reduce inequities in all populations globally [3].

**Fig. 1:**
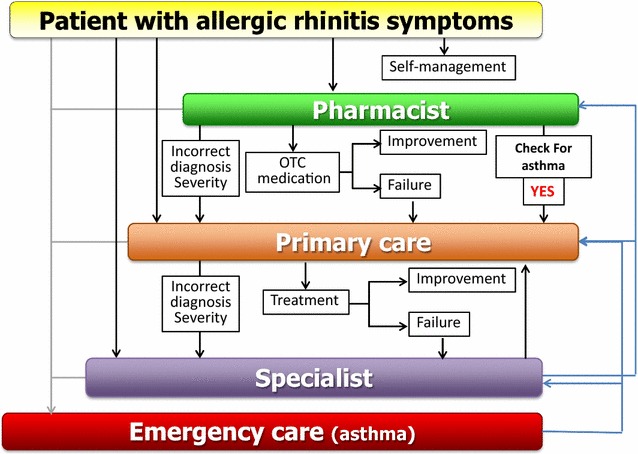
Care pathways for rhinitis (from Bousquet et al. [2, 3])

The initiative Allergic Rhinitis and its Impact on Asthma (ARIA) commenced during a World Health Organization (WHO) workshop in 1999 [6]. It was developed as a guideline [7] using the Grading of Recommendations Assessment, Development and Evaluation (GRADE) approach [8–14]. MASK, the new ARIA initiative, focusses on: (1) the implementation of multi-sectoral care pathways; (2) deploying emerging technologies; (3) with real world data; (4) to provide individualized and predictive medicine; (5) in patients with rhinitis and asthma multi-morbidity; (6) to be used by a multi-disciplinary group or by patients themselves (self-care) using the AIRWAYS ICPs algorithm (Fig. 1); (7) across the life cycle [15, 16].

An App (Android and iOS) [16] has been developed and is associated with an inter-operable tablet for physicians and other healthcare professionals (HCPs) [17]. An elegant and simple common language, the Visual Analogue Scale (VAS), is used to assess and manage AR [18, 19]. It is currently being combined with allergen and pollution exposure using various methods including Google Trends [20, 21]. MASK also includes EQ-5D [22, 23] and CARAT.

The European Commission is co-funding a large collaborative project named JA-CHRODIS (Joint Action on Chronic Diseases and Promoting Healthy Ageing across the Life Cycle) in the context of the 2nd EU Health Programme 2008–2013 [24]. JA-CHRODIS has developed a check-list of 28 items for the evaluation of Good Practices (GP) (http://chrodis.eu/our-work/04-knowledge-platform/). According to the JA-CHRODIS “A GP is not only a practice that is good, but a practice that has been proven to work well and produce good results, and is therefore recommended as a model. It is a successful experience, which has been tested and validated, in the broad sense, which has been repeated and deserves to be shared so that a greater number of people can adopt it.” (http://www.fao.org/docrep/017/ap784e/ap784e.pdf; http://eurohealthnet.eu/sites/eurohealthnet.eu/files/CHRODIS_Promotion%20Material%20WP5-08.pdf).

## Aim of the present paper

MASK is one of the GPs of SUNFRAIL (Reference Sites Network for Prevention and Care of Frailty and Chronic Conditions in community dwelling persons of EU Countries, www.sunfrail.eu), an EU project which evaluates GPs on chronic diseases. The aim of the present paper is to report the results of evaluation performed by using the JA-CHRODIS check-list on MASK. The paper has been devised and written by an expert group including MASK and SUNFRAIL experts.

## CHRODIS check list

1. Equity
  - Design

**Table:** 

Box 1: In design, relevant dimensions of equity are adequately taken into consideration and are targeted (i.e. gender, socioeconomic status, ethnicity, rural–urban area, vulnerable groups)	

People with low socioeconomic status bear a disproportionate burden of allergic diseases. The MASK Action Plan was devised by a group of 450 members. Activities are proposed in a logical framework ranging from existing tools to novel information communication technology (ICT) tools and innovative health systems in order to provide an integrated solution for AR and asthma multi-morbidity across the life cycle. The model of MASK can then be adapted for the health promotion of AHA and management of CRDs across the lifecycle [25]. All dimensions of equity are considered in MASK:

- AR and asthma are life-long diseases often starting early in life. Regional organisations engaged in the EIP on AHA and holding Reference Site status as a result of the 2016 call obtained a grant to facilitate the transfer of innovative practices (Twinning) for implementation in other regions (http://www.scale-aha.eu/news.html). MASK is involved in a Twinning of 24 Reference Sites to better understand and manage AR and asthma in old age adults [26].
- There are gender differences across the lifecycle.
- There are urban and rural differences (users of the App are geolocalized).
- Different socio-economic statuses are assessed in the Twinning.
- The action plan envisions the use of ICT as enablers to address the inequality of allergic diseases on persons with low socioeconomic status by making the tool freely available to all.

Moreover, MASK proposes a common framework of integrated care pathways (ICPs) to facilitate comparability and trans-national initiatives targeted to all populations according to culture, health systems and income [27].

(b) Implementation

**Table:** 

Box 2: In implementation, specific actions are taken to address the equity dimensions	

AIRWAYS ICPs has developed a strategy for low-resource settings based on existing WHO initiatives such as the WHO Package of Essential Non-communicable disease (PEN [28]) or validated primary management strategies in low- and middle-income countries (LMICs) [29]. The first ARIA workshop report (1999) already had a specific goal to reach patients in LMICs [6]. The expertise of GARD for the deployment of GPs in LMICs is used to address the equity dimensions of MASK. Gender is also considered in ARIA.

- Practice
- Comprehensiveness of the intervention

**Table:** 

Box 3: The intervention has a comprehensive approach to health promotion addressing all relevant determinants (e.g. social determinants) and using different strategies (e.g. setting approach)	

Health promotion is an essential component of GARD and MASK. It is extremely important in AR, particularly for the avoidance of allergen, indoor and outdoor air pollution. Google Trends results will be included next year to inform users of the pollen season. A research project using the App has been initiated to determine the impact of air pollution in AR (POLLAR). Patient empowerment is an essential component of MASK and follows the conclusions of the EU Council of the Polish Presidency [30, 31]. Social determinants are considered in the Twinning [26].

WHO defines a setting as “the place or social context in which people engage in daily activities in which environmental, organizational, and personal factors interact to affect health and wellbeing” [32]. The goal of the settings approach is to create supportive environments for optimal health [33]. The model’s key principles include flexibility, community participation, partnership, empowerment and equity [32]. One aspect of the current settings approaches of MASK is to improve work and school productivity [34]. Schools have long been used as a setting to provide health services and, in the future, improvement of school performance and exams may be achievable.

**Table:** 

Box 4: An effective partnership is in place (e.g. multidisciplinary, inter-sector, multi-/and alliances)	

MASK activities are being implemented by a group of 450 members in 70 countries. All stakeholders needed for the implementation of an action plan at the national and local levels actively participate. ARIA has a specific module for pharmacists [35]. Members also include those of previous initiatives such as ARIA [36] and GARD [4, 37]. The majority of members have been working together since 1999. The GA^2^LEN (Global Allergy and Asthma European Network, FP6) network of excellence centres of allergy and asthma [38], EUFOREA (European Forum for Research and Education in Allergy and Airway Diseases) [39, 40] and members of EIP on AHA commitments for action are also involved. Scientific societies participate in the project as well.

**Table:** 

Box 5: The intervention is aligned with a policy plan at local, national, institutional and international levels	

The intervention is linked with WHO (GARD research demonstration project), the EU (DG Research [41–43], DG CONNECT and DG Santé [44]), National Plans (e.g. Finnish Allergy Plan) [45, 46] and EIP on AHA Reference Sites. ARIA is used by the European Medicines Agency (EMA) and the Australian Medical Agency for the labelling of AR interventions.

(b) Description of the practice

**Table:** 

Box 6: The design is appropriate and built upon relevant data, theory, context, evidence and previous practice including pilot studies	

The practice implements (1) multi-sectoral care pathways (2) using emerging technologies (3) with real world data (4) for individualized and predictive medicine (5) in rhinitis and asthma multimorbidity, (6) by a multi-disciplinary group or by patients themselves (self-care) using the AIRWAYS ICPs algorithm (7) across the life cycle [16, 47].

The practice is built on the 19-year scientific basis and experience of stakeholders of the ARIA Working Group [2, 3, 6, 7, 15, 16, 36, 37, 48–53]. Guidelines supporting MASK are based on the GRADE approach and have been developed at McMaster University [8–14, 54].

MASK was initiated by the WHO Collaborating Centre for Asthma and Rhinitis in 2011 and the pilot phase has been completed [8, 12, 13, 17, 36, 55]. MASK proposes to study the symptoms (rhinitis, conjunctivitis and asthma) and work productivity of patients suffering from allergic symptoms, in particular during the pollen season. Geolocalized users assess their daily symptom control using the touchscreen functionality on their smart phone to click on 5 consecutive VAS measures (VAS-global, VAS-nasal, VAS-ocular, VAS-asthma and VAS-work) and type(s) of treatment used.

A clinical decision support system has been finalized based on an ARIA consensus report [47] and digitalized on tablets for HCPs [17]. A care pathway from the patient to the health care professional has been built. It is currently being combined with Google Trends to assess pollen seasons [20, 21], pollen levels and pollution data.

The application is freely available in 17 languages from the Apple App store (iOS) and Google Play Store (Android) in 22 countries (translated and back-translated, culturally adapted and legally compliant). Due to the simplicity of the tool, it can be used in developed and many developing countries (if a smart phone is available).

A pilot study in 5000 users across 20 countries has been analysed. A simple questionnaire administered by cell phones has enabled the identification of phenotypic differences between a priori defined rhinitis groups. The results of the study suggested novel concepts and research questions in AR that cannot be identified using classical methods [56]. A cross-sectional study evaluated the impact of uncontrolled rhinitis assessed by VAS on work productivity using cell phone data collection. It also compared the impact of asthma, rhinitis and conjunctivitis on work [34]. In users with uncontrolled rhinitis, approximately 90% had some work impairment and over 50% had severe work impairment. This pilot study provided not only proof-of-concept for data on the work impairment collected with the app but also data on the app itself, especially the distribution of responses for the VAS. This supports the interpretation that persons with rhinitis report both the presence and the absence of symptoms (submitted). The results of the treatments reported by users may represent a breakthrough in the management of CRDs (in preparation).

*Aiding risk stratification in chronic disease patients with a common strategy*, AIRWAYS ICPs has developed a simple stratification algorithm for asthma control and severity (following a 2009 WHO meeting) which can be extended to all chronic diseases unifying the classification of the diseases for clinical, research and public health use [49, 57, 58].

**Table:** 

Box 7: The design describes the practice in terms of purpose, SMART objectives, methods (e.g. recruitment, location of intervention, concrete activities), and timeframe (sequence, frequency and duration)	

The MASK approach is following SMART objectives:

- *Specific*—Target a specific area for improvement (AR and multi-morbid asthma, addressing symptoms, medication and quality of life).
- *Measurable*—Quantify an indicator of progress (EQ-5D, VAS measures, work productivity).
- *Agreed upon*—Healthcare professionals, policy makers and patients.
- *Realistic*—Objectives are achievable. Results are already obtained for the pilot study, given available resources. There are over 11,000 users.
- *Time*-*related*—Results are available and a plan with objectives fixed for 2017 and 2018 is in place.

The methods are clearly stated and published [16].

- Recruitment: The *Allergy Diary* was used by people who downloaded it from the Apple App store, Google Play store, and other Internet sources. A few users were clinic patients that were asked by their physicians to access the app. Due to anonymization (i.e. no name or address) of data, no personal identifiers were gathered. None of the users were enrolled in a clinical study as we aimed to have a real life assessment. There was no specific advertisement or other recruitment campaign.
- The *Allergy Diary* collects information on allergic symptoms.

3. Ethical considerations

**Table:** 

Box 8: The intervention is implemented equitably, i.e. proportional to needs	

The potential for inequities arising from the use of MASK has been considered. MASK might raise legal and ethical questions in employment (work productivity) or access to private insurance. However, users are anonymized. The freely-available App increases accessibility for vulnerable groups, although concerns on the digital divide should be addressed [59]. The application requires a smart phone, which limits its universal access at the moment. Notwithstanding, the authors consider that the information obtained from the current smart phone users will benefit future users, in a progressively higher number.

**Table:** 

Box 9: Potential burdens, including harm, of the intervention for the target population are addressed	

ICT can improve health outcomes, quality of life and efficiency of health care processes but may also contain disruptive consequences. Moreover, the implementation of ‘e-health applications’ is rather complicated. E-health applications do not (often) provide direct benefit that can be easily measured [60]. Incentivising further technological development without putting enough emphasis on and properly supporting, even financially, its adoption is likely to widen the serious ‘technology consumption gap’ that we all witness [61]. Nevertheless, mobile phones are widely used among populations with poor access to health care and limited education. They provide the opportunity to disseminate relevant information and empower individuals for guided self management of diseases. MASK is currently investigating these aspects.

**Table:** 

Box 10: The intervention’s objectives and strategy are transparent to the target population and stakeholders involved	

The Terms of Use [56] have been translated into all relevant languages and customized according to the legislation of each country in order to allow the use of the results for research purposes.

The data were anonymized except for geolocalized data to the area-level [56]. The European Commission’s Article 29 Working Party states that geolocation information is personal data (http://ec.europa.eu/newsroom/just/item-detail.cfm?item_id=50083) and that information can be collected, shared, or stored only with the express consent of the individual. This is the case for MASK because users agree to geolocation in the terms of use of the App. Moreover, geolocation is optional and each user can allow it or not on his/her cell phone. Geolocation, if active, can be disallowed at any time. Finally, geolocation is not used in the data mining process and the phone IP is not retained.

Formal Institutional Review Board (IRB) approval was not required for the first two studies. An IRB approval has been requested for the Twinning. Although registered as CE1, the App is considered as a non-medical device by the MHRA (Medicines and Healthcare products Regulatory Agency, UK Government, www.gov.uk/government/organisations/medicines-and-healthcare-products-regulatory-agency) and by the Ethics Committee of Cologne University.

4. Evaluation

**Table:** 

Box 11: There is a defined and appropriate evaluation framework assessing structure, process and outcomes. The use of validated tools and/or the results of evaluation are linked to actions to reshape the implementation accordingly and/or the intervention is assessed for efficiency (cost vs. outcome)	

MASK data are available using a real-time database and results are regularly published. Some 2016 data are already in press [34, 56].

The results of 2016 have induced a change in some of the questions of the App and in the re-analysis of data using a novel approach, which suits observational studies better. In randomized controlled trials (RCTs), each subject is randomly assigned to a treated or control group, whereas observational studies examine the possible effect of a treatment on subjects where the investigator has no control over the experiment and cannot randomize the allocation of subjects [62]. This can create bias, may mask cause and effect relationships or, alternatively, suggest incorrect correlations. However, observational studies reflect “real world” use and practice more closely than RCTs in terms of the heterogeneous patient populations included and the variety of medical interventions [63]. They can provide clinically-relevant information, not necessarily provided by RCTs. Given the limitations of an observational study approach, it is important to optimize their study design to maximize their validity. In particular, known causes of bias and confounding should be measured [63].

The Twinning questionnaire in the 24 Reference Sites has been updated based on 2016 data [26].

One of the major goals of MASK is to improve loss of work productivity due to AR. Costs range from 30 to 60 B€ a year in Europe [64]. The pilot study allowed us to show that MASK can accurately assess work productivity [34]. In the Twinning, work productivity will be assessed in several settings including the Northern Ireland NHS, North of England and Valencienne, France hospitals.

EQ-5D is one of the MASK tools and will make it possible to assess the cost-effectiveness of interventions. However, a sufficient amount of data is needed and results are expected in 2018.

**Table:** 

Box 12: Evaluation results achieve the stated goals and objectives	

The results of ARIA are clear, but the results of MASK can only be assessed and its impact understood when a sufficient number of users will have been monitored. Interim data from pilot studies are encouraging [26, 34, 56]. They show in 11,300 users (June 17) that the phenotype of AR can be assessed and some features such as work productivity can be appraised.

**Table:** 

Box 13: Evaluation Information/monitoring systems are in place to regularly deliver data aligned with evaluation and reporting needs	

A real-time database is available and the statistical analysis of the data is in place, allowing for a few preliminary reports to be published or in preparation already.

**Table:** 

Box 14: The intervention is assessed for outcomes, intended or unintended	

Outcomes measured by MASK include not only multiple symptoms but also EQ-5D and work productivity assessment.

5. Empowerment and participation

**Table:** 

Box 15: The intervention develops strengths, resources and autonomy in the target population(s) (e.g. assets-based, salotogenic approach)	

MASK focusses on factors that support human health and well-being, rather than on factors that cause disease. This “salotogenic approach” is concerned with the relationship between health, stress, and coping [65]. AR is particularly suited for the model since AR is not a lethal disease, does not lead to ED visits or hospitalizations but has a major impact on quality-of-life [66, 67], school and work [34, 68–78]. The evolutionary nature of the development of the app allows for continual assessment of both recording and intervention. This offers the potential for the technological intervention, always blended with clinician input, to induce behavioural change in patients to improve their outcomes [79]. Furthermore, MASK may provide a model for evaluating feasibility and effectiveness of using mobile technology for empowering individuals for diagnosis, early recognition of worsening of their diseases and guided self management of chronic diseases.

**Table:** 

Box 16: The intervention achieves meaningful participation among the intended target population	

In order to assess the participation of the target population, a qualitative study has been carried out in users in France (MADoPA, http://www.madopa.fr/). The results of the study are under evaluation and we plan to extend the study to the European population to better understand the participation of the target population. The preliminary data of the study indicated that users were willing to show their data to their physician. A new functionality has therefore been added (March 1, 2017) allowing patients to print their data. There is no direct link from the patient’s cell phone to the physician’s computer (to comply with regulations).

**Table:** 

Box 17: The intervention is designed and implemented in consultation with the target population	

Quality of life—normal life despite the disease, cure and prevention—represents the patient’s goal in AR and asthma [31]. Patient perspective, represented by patients’ organizations, arises from the collective experience of patients (as well as their parents and partners) living with allergy. The European Federation of Allergy and Airways Diseases Patients’ Associations (EFA, http://www.efanet) is an alliance of 41 allergy, asthma and chronic obstructive pulmonary disease (COPD) patients’ organizations in 25 countries. Patient perspective has been incorporated at all levels of ARIA, GARD and MASK, from the early steps.

The goal and rationale of patient involvement in medical decisions is patient empowerment [31]. Empowered patients know their disease, have the skills and motivation to take good care in their everyday life, adjust treatment, are prepared in new or potentially exacerbating situations, detect side-effects, contact a healthcare professional when needed and adhere to treatment regime. Many tools support empowerment, shared decision making models and patient education. Another key aspect of patient involvement in medical decisions is the involvement of patient representatives in the healthcare policy and organization in practice. The members of EFA have developed tools to help in involvement in medical decisions and empowerment. These tools were acknowledged while developing MASK.

6. Target population

**Table:** 

Box 18: Target populations are defined on the basis of needs assessment including strengths and other characteristics	

Patients, clinicians and other HCPs are confronted with various treatment choices for the management of AR. This contributes to considerable variation in clinical practice and patients are often unsatisfied by their treatment. Severe Chronic Upper Airway Disease (SCUAD) defines uncontrolled AR patients despite optimal pharmacotherapy [80] and accounts for 10–20% of patients receiving treatment for AR [81]. A large number of AR patients appear to be self-managing their condition with few interactions with their doctor regarding their allergy prescription [82]. Many AR patients use over-the-counter (OTC) drugs [35, 83, 84] and only a fraction have had a medical consultation. The vast majority of patients who visit GPs or specialists have moderate/severe rhinitis [85–87]. A large number of OTC or prescribed drugs are available for the patient who can also choose alternative medicine or allergen-specific immunotherapy [88]. The app will also be useful in the early identification of those who are unaware of being affected by allergic rhinitis, and of the fact that symptoms can be controlled. The MASK ICPs consider a multi-disciplinary approach including self-management as proposed by AIRWAYS ICPs [2]. In the *Allergy Diary*, both OTC and prescribed medications are listed and the list has been customized for each country.

**Table:** 

Box 19: The engagement of intermediaries/multipliers is used to promote the meaningful participation of the target population	

A transfer innovation is ongoing from the App developed by the MACVIA-France EIP on AHA reference site (*Allergy Diary*) to 25 Reference Sites or regions across Europe. Validated ICT tools (*Allergy Diary* and CARAT: Control of Allergic Rhinitis and Asthma Test) are being used [26].

7. Sustainability

**Table:** 

Box 20: The continuation of the intervention is ensured through institutional ownership that guarantees funding and human resources	

MASK belongs to the Fondation Partenariale FMC VIA LR of the French Ministry of Education and Research (*Fondation des maladies chroniques et du vieillissement actif*—*Languedoc*-*Roussillon*, *NOR: MENS1500573A, arrêté du 9*-*9*-*2015, MENESR*—*DGESIP B1*-*3*). The four partners of the Foundation are the University of Montpellier, the Région Occitanie, and the University hospitals of Montpellier and Nîmes. There is a guarantee for the funding and human resources needed to accomplish the project.

However it should be noted that sustained engagement by individual patients is not necessarily a measure of success. For example, patients with intermittent allergic rhinitis may choose to inform MASK only at specific times of the year.

**Table:** 

Box 21: There is broad support for the intervention amongst those who implement it	

There are 450 stakeholders in the MASK working groups, from over 70 countries and all continents. They represent all groups from patients to policy makers, practicing health care professionals and key opinion leaders.

**Table:** 

Box 22: There is broad support for the intervention amongst the intended target populations	

This has not yet been evaluated. It is planned for 2017 by the Twinning [26].

8. Governance

**Table:** 

Box 23: The intervention includes an adequate estimation of the human resources, material and budget requirements in clear relation with committed tasks	

See above (Box 20). Initial seed funding has been donated from private partners (Unrestricted educational grant) as well as from EU Structural and Development Funds. The private sector donors did not participate in any decision on the project. Funding for 2018 is secured. A business plan has been set up by the SME (small and medium enterprise) Kyomed (Montpellier) and by EUFOREA (Belgian ASBL).

**Table:** 

Box 24: Sources of funding are specified in regards to stability and commitment	

See Box 23.

**Table:** 

Box 25: Organisational structures are clearly defined and described (i.e. responsibility assignments, flows of communication and work as well as accountabilities)	

These were the first steps of the MASK project. The *Fondation Partenariale “Fondation des maladies chroniques et du vieillissement actif*—*Languedoc*-*Roussillon*” was established to coordinate the actions of the MACVIA-France Reference Site [89] and to lead the MASK project (see Box 20).

9. Scalability

**Table:** 

Box 26: Potential impact on the population targeted (if scaled up) is assessed	

MASK focuses on factors that support human health and well-being, as well as on factors that cause disease (“salutogenic approach” [65]). An important outcome of MASK is work productivity. The same applies for school learning as AR has a detrimental effect on learning and on the results of exams [90]. Quality-of-life is tested using EQ-5D.

One major problem of all allergic diseases is compliance with treatment. If appropriately theorized and developed, ICT solutions are expected to improve compliance possibly by inducing behavioural change, and therefore the control of AR and asthma [91]. However, this component has not yet been tested in MASK.

**Table:** 

Box 27: There is a specific knowledge transfer strategy in place (evidence into practice)	

The scaling up strategy uses the recommendations of the European Innovation Partnership on Active and Healthy Ageing [51]. The overarching goals of the MASK approach are to provide an active and healthy life to rhinitis sufferers, whatever their age, sex or socio-economic status, in order to reduce health and social inequalities incurred by the disease.

*Scaling*-*up strategies in Europe and beyond* The scaling up strategy has been clearly defined and approved by AIRWAYS ICPs members. It follows the EIP-AHA recommendations on a 5-step framework: [1] what to scale up: (1–1) databases of Good Practices, (1–2) assessment of viability of the scaling up of Good Practices, (1–3) classification of Good Practices for local replication; and [2] how to scale up: (2–1) facilitating partnerships for scaling up, (2–2) implementation of key success factors and lessons learnt, including emerging technologies for individualised and predictive medicine. Scaling-up will take place within and beyond Europe with GARD [25, 30, 31, 45, 92].

MASK is implemented in 22 countries (17 languages). We aim to include five more countries in 2017.

*Strengthening the WHO NCD Action Plan* AIRWAYS ICPs is a GARD demonstration project (WH0). It is in line with the WHO NCD Action Plan since it aims to reduce the preventable and avoidable burden of morbidity, mortality and disability by means of multi-sectoral collaboration and cooperation at national, regional and global levels.

**Table:** 

Box 28: An analysis of requirements for eventual scaling up such as foreseen barriers and facilitators (e.g. resources, organisational commitment, etc.) is available	

We use the expertise of WHO GARD to overcome generic barriers that may impact scaling up. Moreover, in each country, a MASK working group is in place to overcome local barriers.

10. Assessment of the criteria using SurveyMonkey

A SurveyMonkey questionnaire is the easiest way to create surveys and to obtain answers. It allows a survey to be prepared quickly and targeted answers to be obtained from the audience requested. We conducted a SurveyMonkey (www.surveymonkey.com) of expert clinical opinion on the 28 items to assess the robustness of the answers to the boxes. The SurveyMonkey was sent to 34 experts from different fields. For each item, respondents indicated their level of agreement on a VAS ranging from 0 (strongly disagree) to 100 (strongly agree). 18 experts from 12 countries responded (53%). It was decided pre-hoc to stop the survey when a 50% response was achieved. Respondents included allergists, general practitioners, pharmacologists, respiratory physicians or Public Health professionals. Two of the experts did not disclose their affiliation. Five SUNFRAIL experts were included in the survey. We categorized a priori the level of response (in line with the checklist ≥ 80, partly in line with the checklist: 50–79 and not in line with the checklist < 50).

**Fig. 2:**
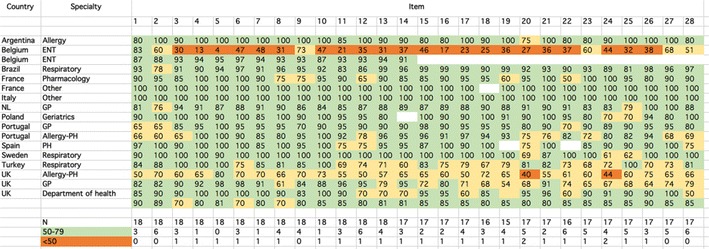
Results of the SurveyMonkey

The results are presented in Fig. 2. Most experts agreed that most items were in line with the CHRODIS checklist. However, one ear, nose and throat (ENT) expert from Belgium and one Allergy-Public health expert from the UK found that many items were not partly or not in line with the checklist. Overall, from 75 to 94% of items were found to be in line with the CHRODIS checklist.

Comments are provided for a percentage of agreement < 75%.

### Box 2

At this stage there is no change in MASK incurred by the SurveyMonkey. There will be a revision of the product at the end of 2017 taking the comments into consideration.

### Box 8

Since the SurveyMonkey, ethical committee has been granted (Kohln-Bohn Region) and the MHRA and the ethics committee have indicated that the App is not a medical device.

### Box 12

More data have been evaluated and there is a strong consistency of results.

### Box 13

At this stage there is no change in MASK incurred by the SurveyMonkey. There will be a revision of the product at the end of 2017 taking the comments into consideration.

### Box 17

At this stage there is no change in MASK incurred by the SurveyMonkey. There will be a revision of the product at the end of 2017 taking the comments into consideration.

### Box 18

At this stage there is no change in MASK incurred by the SurveyMonkey. There will be a revision of the product at the end of 2017 taking the comments into consideration.

### Box 19

The transfer of innovation is increasing. It is already implemented in Australia, Brazil, Mexico, and is in process in Argentina, Paraguay and Uruguay. Novel approaches including air pollution and allergen exposure are being considered and should be available by the end of 2018.

### Box 20

New private funding has been secured. Moreover, the continuation of the intervention is ensured through institutional ownership.

### Box 22

At this stage there is no change in MASK incurred by the SurveyMonkey. There will be a revision of the product at the end of 2017 taking the comments into consideration.

### Box 23

See Box 20.

### Box 24

See Box 20.

### Box 25

At this stage there is no change in MASK incurred by the SurveyMonkey.

### Box 27

At this stage there is no change in MASK incurred by the SurveyMonkey.

### Box 28

At this stage there is no change in MASK incurred by the SurveyMonkey. There will be a revision of the product at the end of 2017 taking the comments into consideration.

## Conclusions

MASK assessment using The European Union Joint Action JA-CHRODIS (Joint Action on Chronic Diseases and Promoting Healthy Ageing across the Life Cycle) checklist of 28 items for the evaluation of Good Practices by a panel of experts from several countries indicates that it is in line with the CHRODIS recommendations.

## Appendix 1: Terms of use for the UK

By installing and using this app, you agree to the applicability of these Terms of Use (and to the privacy policy below). If you do not agree to these Terms of Use, you must not download or use this app, copyright of which is protected.

1. This app provides a digital way for you to keep a diary with respect to the symptoms of an allergy. The diary gives you insight into your allergies by enabling you to ‘score’ your allergy-related symptoms and view your results in a graph. An advice text is shown together with the results and you may be advised to see a healthcare provider.
2. This app is intended for general information purposes and must not be used as a substitute for any kind of professional and/or medical advice.
3. This “Allergy diary App by MACVIA-ARIA” is based on previous recommendations and the ARIA^®^ guidelines (2015) with permission of ARIA^®^. Its diagnostic and therapeutic uses should be carried out in conjunction with patients’ physicians. The ARIA^®^ guidelines have been published in the peer-reviewed medical literature and have been reviewed and approved by medical experts worldwide. The use of this application in all countries should be carried out only with the specific therapies that are licensed in that country. MACVIA and ARIA will be held harmless for any inappropriate use of this application. Users of the application should read, understand and follow the approved prescribing information for any medications used in conjunction with the application.
4. This app is provided to you for your personal use and you must not use it to process the personal information of any other person.
5. This app, and the information contained in it, is provided to you “as is,” for informational purposes only, with no guarantee of completeness, accuracy, timeliness or of the results obtained from the use of this information, and without warranty of any kind, express or implied, including but not limited to warranties of performance, merchantability and fitness for a particular purpose.
6. You recognise that the app functionality may be affected by changes in the operating system of your device. It is your responsibility to check for updates in order to determine if you have the most current version of the app, to ensure that it is working properly.
7. Neither Peercode or MACVIA-ARIA, nor its subsidiaries, affiliates and/or respective employees or management, is liable for any damages, including, and without limitation, direct, indirect, consequential or incidental damages resulting from or in connection with any information, materials, qualifications or recommendations related to this app.
8. Peercode/MACVIA-ARIA is not liable for any damages resulting from the use (or inability to use) this app, including damages caused by viruses or any incorrectness or incompleteness of the Information. Peercode/MACVIA-ARIA is also not liable for damages resulting from the use of electronic devices for communication with this app, including—but not limited to—damages resulting from failure or delay in delivery of electronic messages, interception or manipulation of electronic messages by third parties or by software/hardware used for electronic communications and transmission of viruses.
9. If this app directly or indirectly provides links to other information, such as information on websites maintained by third parties over whom Peercode/MACVIA-ARIA has no control, Peercode/MACVIA-ARIA is not liable for the use or content of this information.
10. Peercode/MACVIA-ARIA reserves the right to change the Allergy Diary App Terms of Use at any time and without notice.
11. These Terms of Use shall be governed by and construed in accordance with the laws of the Netherlands. The competent court in Amsterdam shall settle all disputes arising in connection with these terms.

- *Contact information*
- Peercode may be contacted via:
- Address: Oudenhof 4, 4191 NW Geldermalsen,
- Country: The Netherlands
- Phone: +31 (0)88 0084100

## Abbreviations

AHA: active and healthy ageing
AIRWAYS ICPs: integrated care pathways for airway diseases
AR: allergic rhinitis
ARIA: allergic rhinitis and its impact on asthma
CARAT: control of allergic rhinitis and asthma test
COPD: chronic obstructive pulmonary disease
CRD: chronic respiratory diseases
DG: directorate general
DG CONNECT: Directorate General for Communications Networks, Content & Technology
DG Santé: Directorate General for Health and Food Safety
EFA: European Federation of Allergy and Airways Diseases Patients’ Associations
EIP on AHA: European Innovation Partnership on AHA
EMA: European Medicines Agency
ENT: ear, nose and throat
EQ-5D: Euroquol
GARD: WHO global alliance against chronic respiratory diseases
GP: Good Practice
GRADE: grading of recommendations assessment, development and evaluation
HCP: health care professional
ICP: integrated care pathway
ICT: information and communication technology
IRB: Institutional Review Board
JA-CHRODIS: joint action on chronic diseases and promoting healthy ageing across the life cycle
LMICs: low- and middle-income countries
MACVIA-LR: contre les MAladies Chroniques pour un VIeillissement Actif (Fighting chronic diseases for AHA)
MASK: MACVIA-ARIA Sentinel NetworK
MeDALL: Mechanisms of the Development of ALLergy (FP7)
MHRA: Medicines and Healthcare products Regulatory Agency
NCD: non-communicable disease
OTC: over-the-counter
PEN: package of essential noncommunicable disease
RCT: randomized controlled trial
SCUAD: severe chronic upper airway disease
SUNFRAIL: Reference Sites Network for Prevention and Care of Frailty and Chronic Conditions in community dwelling persons of EU Countries
VAS: visual analogue scale
WHO: World Health Organization
